# Gender-Related Determinants of Adherence to the Mediterranean Diet in Adults with Ischemic Heart Disease

**DOI:** 10.3390/nu12030759

**Published:** 2020-03-13

**Authors:** Valeria Raparelli, Giulio Francesco Romiti, Valeria Spugnardi, Marco Borgi, Roberto Cangemi, Stefania Basili, Marco Proietti, the EVA Collaborative Group

**Affiliations:** 1 Department of Experimental Medicine, Sapienza University of Rome, 00161 Rome, Italy; 2 Department of Translational and Precision Medicine, Sapienza University of Rome, 00161 Rome, Italy; giuliofrancesco.romiti@uniroma1.it (G.F.R.); valeria.spugnardi@gmail.com (V.S.); marco.borgi9@gmail.com (M.B.); roberto.cangemi@uniroma1.it (R.C.); stefania.basili@uniroma1.it (S.B.); 3 Department of Clinical Sciences and Community Health, University of Milan, 20122 Milan, Italy; marco.proietti@unimi.it; 4 Geriatric Unit, Fondazione IRCCS Cà Granda, Ospedale Maggiore Policlinico, 20122 Milan, Italy; 5 Liverpool Centre for Cardiovascular Science, University of Liverpool and Liverpool Heart & Chest Hospital, Liverpool L7 8TX, UK

**Keywords:** Ischemic heart disease, Mediterranean diet, sex, gender, adherence

## Abstract

Background: The reasons behind low adherence to the Mediterranean diet (Med-diet) are still not entirely known. We aimed to evaluate the effect of biological (i.e., sex-related) and psycho-socio-cultural (i.e., gender-related) factors on Med-diet adherence. Methods: Baseline Med-diet adherence was measured using a self-administered questionnaire among adults with ischemic heart disease (IHD) from the EVA (Endocrine Vascular Disease Approach) study. A multivariable analysis was performed to estimate the effect of sex- and gender-related factors (i.e., identity, roles, relations, and institutionalized gender) on low adherence. Results: Among 366 participants (66 ± 11 years, 31% women), 81 (22%) adults with low adherence demonstrated higher rates of diabetes, no smoking habit, lower male BSRI (Bem Sex Role Inventory) (median (IQR) 4.8 (4.1 to 5.5) vs. 5.1 (4.5 to 5.6) and *p* = 0.048), and higher Perceived Stress Scale 10 items (PSS-10) (median (IQR) 19 (11 to 23) vs. 15 (11 to 20) and *p* = 0.07) scores than those with medium-high adherence. In the multivariable analysis, only active smoking (odds ratio, OR = 2.10, 95% confidence interval, CI 1.14 to 3.85 and *p* = 0.017), PPS-10 (OR = 1.04, 95% CI 1.00 to 1.08, and *p* = 0.038) and male BSRI scores (OR = 0.70, 95% CI 0.52 to 0.95, and *p* = 0.021) were independently associated with low adherence. Conclusions: Male personality traits and perceived stress (i.e., gender identity) were associated with low Med-diet adherence regardless of the sex, age, and comorbidities. Therefore, gender-sensitive interventions should be explored to improve adherence in IHD.

## 1. Introduction

Diet is one of the most important lifestyle behaviors which influences the development of many pathological conditions [1]. Thus, European guidelines on cardiovascular disease (CVD) prevention recommend a healthy and balanced diet, consisting of a high intake of monounsaturated fatty acids, fiber, fruits, vegetables, fish, seeds, nuts, and a limited consumption of alcohol, (red) meat, dairy products, and saturated fatty acids [2].

The Mediterranean diet (Med-diet) [3] is well studied and has been shown to strongly impact health-related outcomes, with reduced morbidity and mortality, especially for cardiovascular and cerebrovascular events [4]. The Med-diet fits CVD prevention guidelines, as it is characterized by a high amount of fruits, vegetables, legumes, whole grains, nuts, and monounsaturated fatty acids (especially extra virgin olive oil), along with a moderate intake of fish, white meat, and red wine. Finally, the Med-diet includes low consumption of red meat, dairy products, and saturated fatty acids [3].

Despite the well-known beneficial effects and the protective role for cardiovascular diseases, a low adherence rate to the Med-diet has been recently documented, especially in the Mediterranean countries themselves [4,5,6]. Multiple factors have been identified as predictors of poor adherence to the Med-diet, including female sex, obesity, and diabetes [7]; however, most of this evidence is derived from relatively small sample-size populations and from specific geographical settings [8,9,10]. Furthermore, these studies have are typically focused on classical, biological causes of poor adherence.

Biological sex and gender-related (i.e., psycho-socio-cultural) factors play an important role as health determinants. Despite their importance, however, both aspects are generally overlooked and underreported [11,12,13,14,15,16]. Specifically, gender represents a complex construct, which includes the behavioral, cultural, environmental, and social characteristics of the individual [11,17]. Gender encompasses the following four domains: (i) identity (i.e., how individuals perceive and present themselves); (ii) roles (i.e., behavioral expectations based on individual sex including role in the family, in the workplace, in society, etc.); (iii) relations (i.e., emotional and economic relations between individuals); and (iv) institutionalized gender (i.e., the distribution of power between individuals in the political, educational, religious, media, medical, and social institutions in any society) [18].

Throughout the last decade, it has progressively become clear to researchers that sex and gender are not independent; therefore, exclusively assessing one or the other fails to account for identified variations in health [11,12]. Within the context of the Med-diet, the impact of their intersectionality as determinants of adherence have not been fully explored. This is mainly due to the lack of a validated standardized measurement of gender to be applied in clinical research, with the exception of the GENESIS-PRAXY gender score [12,17,19,20,21].

We explored the influence of sex- and gender-related factors on the Med-diet adherence based on a prospective cohort of Italian adults hospitalized for ischemic heart disease (IHD) with the unique systematic collection of gender-related variables.

## 2. Materials and Methods

Data for the present analysis comes from the “Endocrine Vascular Disease Approach” (EVA) project (ClinicalTrials.gov identifier NCT02737982), an ongoing observational, prospective study, aimed at exploring sex and gender-related differences in the interaction between platelet function, sex hormone balance, and coronary microvascular dysfunction in IHD. The EVA study design has been previously published [22]. Briefly, EVA is an observational registry of men and women (≥18 years) that were referred to the cardiac catheterization laboratory (cath-lab) to undergo coronary angiography or percutaneous coronary intervention for suspected IHD. Therefore, the EVA population includes individuals with either obstructive or nonobstructive coronary artery disease.

The study was conducted in full conformance with the principles of the Declaration of Helsinki, and was approved by the local Ethics Committee, Sapienza University of Rome, Italy (reference 3786, 24/09/2015). Written informed consent was obtained from all participants and the recruitment phase was ongoing. For the purpose of the current study, we selected participants recruited from April 2016 until August 2019, with access to complete baseline clinical and gender-related characteristics, as well as the evaluation of adherence to the Med-diet.

### 2.1. Baseline Clinical Characteristics and Gender-Related Factors

We identified individuals with the following complete baseline clinical characteristics: (i) physical examination data (i.e., height, weight, blood pressure, and heart rate); (ii) family history of CVD and the presence of traditional risk factors, such as smoking, hypertension, dyslipidemia, and type 2 diabetes mellitus (T2DM); (iii) presence of concomitant diseases such as previous myocardial infarction (MI), heart failure, cerebral vasculopathy (prior stroke or transient ischemic attack, TIA), chronic obstructive pulmonary disease (COPD), and connective tissue disease. For all participants, we defined based on angiographic data, the presence of coronary artery obstruction (<50%: nonobstructive CAD; ≥50% obstructive CAD) and severity of vessel impairment (i.e., number of injured vessels to distinguish mono vessel vs. multivessel diseases). Functional status, assessed by the Duke Activity Status Index [23], and pharmacological therapy at baseline, were also available.

In accordance with the definition of gender domains proposed by the Women’s Health Research Network [18], we collected the following gender-related factors: (i) gender relations, i.e., marital status (i.e., married and living with partner vs. others) and social and emotional support, assessed by the “Enhancing Recovery in Coronary Heart Disease (ENRICHD) Social Support Inventory” (ESSI) questionnaire [24]; (ii) gender roles, i.e., household’s primary earner status and employment; (iii) institutionalized gender, i.e., low education level (i.e., less than secondary school) and low household income (i.e., <1000 Euro per month); and (iv) gender identity, i.e., Bem Sex Role Inventory (BSRI) [25] and level of stress, (Perceived Stress Scale 10 items (PSS-10)) [26].

The BSRI is a self-reported questionnaire that categorizes a respondent’s personality. The questionnaire is based on gender stereotypes (masculine, feminine, or neutral) and measures how well a person fits into traditional sex roles [25].

The PSS-10 is a questionnaire that measures an individual’s self-perception of stress. This questionnaire is based on ten items that explore the individual’s perception of their experienced stress in the past month [26].

Furthermore, we defined the following as risk-taking behaviors: physical inactivity (defined as no recreational activity or less than once per week) and cohabitation with a smoker.

Participants’ characteristics were selected from a combination of medical record abstraction and standardized in-person interviews administered by skilled personnel.

### 2.2. Assessment of Adherence to the Med-Diet

The adherence to the Med-diet was evaluated using a self-administered, previously validated questionnaire at the time of enrollment, consisting of 14 items [27]. (Appendix A, Table A1) A point was assigned for each answer if the frequency of consumption of the food respected the principles of the Med-diet. In particular, 1 point was assigned if olive oil was the main culinary fat, and if the daily consumption was at least 4 tablespoons (60 mL), if the daily consumption of vegetables was at least two portions (where one portion was at least 200 g), and if the daily consumption of fruit or natural fruit juices was at least 3 units. Furthermore, 1 point was awarded if the daily consumption of red meat, butter, margarine or cream and sweetened or carbonated beverages was <1. If at least 7 glasses of wine were consumed during meals within one week, 1 point was given. Similarly, if at least 3 portions of legumes and fish or shellfish were consumed in a week, one point was awarded. One point was assigned if commercial sweets or pastries (not homemade) were consumed less than 3 times a week, if the daily consumption of carbonated beverages was less than one and if the consumption of nuts (including peanuts) was at least once a week. Finally, 1 point was awarded if the participant preferred to eat chicken, turkey, or rabbit meat over red meat and if the sofrito (sauce made with tomato and onion, leek or garlic and simmered with olive oil) was used at least twice a week.

A score that was equal to or less than 6, as obtained from the sum of the 14 responses, defined a low Med-diet adherence.

### 2.3. Statistical Analysis

After verifying the normality of the continuous variables, variables found to be normally distributed were reported as mean ± standard deviation (SD), whereas those found to be non-normally distributed were reported as median and interquartile range (IQR). Differences between the groups (low-adherent vs. medium-high adherent) were established with the Student’s T-test and with the Mann–Whitney U-test, where appropriate.

Categorical variables were reported as counts and percentages, and differences between groups (low-adherent vs. medium-high adherent) were evaluated with the Chi-square test or Fisher’s Exact test when appropriate.

A logistic regression model was used to identify predictors and factors associated with low adherence to the Med-diet. Variables which significantly differed at baseline with a *p*-value < 0.10 were included in the final multivariate model. A two-sided *p*-value < 0.05 was considered to be statistically significant.

All analyses were performed using SPSS v. 25.0 (IBM, NY, USA).

## 3. Results

**Figure 1:**
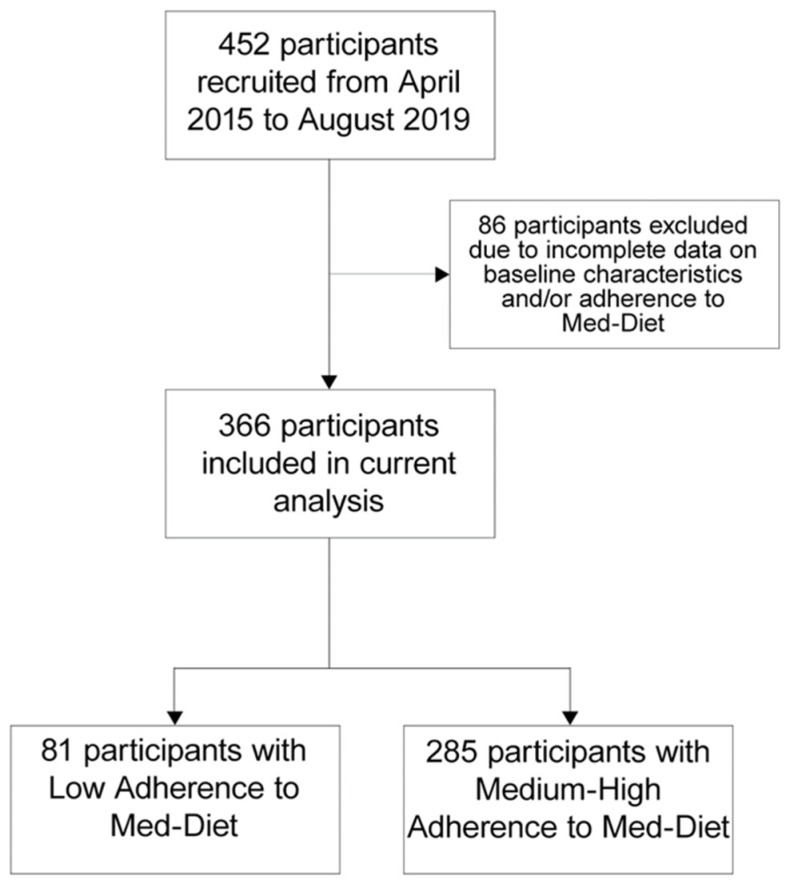
Flow chart of the study.

Four hundred and fifty-two participants were enrolled between April 2016 and August 2019. Within this cohort, 366 (age 66 ± 11 years, 31% women) individuals were included in the present analysis (Figure 1). A low Med-diet adherence was reported in 81 (22%) cases.

Clinical characteristics according to adherence to the Med-diet are reported in Table 1.

Adults with a low Med-diet adherence had a higher prevalence of connective tissue disease (3.7% vs. 0.7% and *p* = 0.04), were more likely current smokers (37.0% vs. 23.2% and *p* = 0.01) and less frequently affected by T2DM (16.0% vs. 27.0% and *p* = 0.04) as compared with the participants with medium-high adherence. No statistically significant differences were observed between subjects with medium-high adherence and subjects with low adherence to the Mediterranean diet with respect to age, sex, BMI, major cardiovascular comorbidities, and lifestyle behaviors, such as physical inactivity, polypharmacy, and DASI.

Angiographic characteristics of EVA patients, according to the adherence to the Mediterranean diet, are reported in Table 2. No statistically significant differences were observed regarding the type of CAD (i.e., nonobstructive vs. obstructive), the number of injured vessels (i.e., mono vessel or multivessels), and the type of clinical presentation (i.e., acute vs. chronic).

The distribution of gender-related factors according to adherence to the Med-diet is shown in Table 3. Adults with low Med-diet adherence had a lower male BSRI than those with medium-high adherence (median (IQR) 4.8 (4.1 to 5.5) vs. 5.1 (4.5 to 5.6) and *p* = 0.048). A low Med-diet adherence was associated with a higher PSS-10 score, although this finding was not statistically significant. The univariate analysis for selected variables is reported in Table 4.

In the multivariate analysis conducted to identify the factors independently significantly associated with the Med-diet, active smoking (odds ratio, OR = 2.10, 95% CI 1.14 to 3.85, and *p* = 0.017), perceived stress (OR = 1.04, 95% CI 1.00 to 1.08, and *p* = 0.038), and the male BSRI (OR 0.70, 95% CI 0.52 to 0.95, and *p* = 0.021) were found to be independently associated with low Med-diet adherence. The model was adjusted for sex, age, and comorbidity (Table 5).

## 4. Discussion

The main finding of our analysis is that gender-related factors are associated with a low adherence to the Med-diet in patients hospitalized for IHD. A low adherence to Med-diet was frequent (about one-fifth), associated with smoking habits and higher levels of perceived stress, as well as inversely related to the presence of male personality traits.

Adherence to the Med-diet has been linked to reduced morbidity and mortality, especially for cardiovascular and cerebrovascular causes, with biological and mechanistic explanations that underlie this relationship [4,28]. However, in recent years, there has been a progressive loss of healthy and beneficial dietary models, such as the Mediterranean dietary pattern [4,5,6]. The mechanisms responsible for the low adherence to the Med-diet, however, remain unknown, and the role of gender-related (i.e., psycho-socio-cultural) factors is particularly underestimated [11]. In light of this, our findings raise important questions about the mechanisms at an individual level that can explain poor adherence to the Med-diet, and more generally, to healthy habits in individuals with high cardiovascular risk, despite their well-known and proven cardiovascular benefits.

Most clinical studies have focused on the biological determinants of adherence, including the presence of comorbidities, such as T2DM [7]. In our cohort, T2DM was the only cardiovascular risk factor whose prevalence was significantly higher in adults with a medium-high adherence to the Med-diet. The American and European guidelines for T2DM management recommend a diet rich in fiber from unrefined vegetables, fruit, and cereals, and poor in animal fats; therefore, the Med-diet ranks among the main recommended dietary patterns [29]. Our findings could reflect the increasing campaign to improve nutritional therapy as a non-pharmacological intervention in individuals with T2DM. In fact, the Med-diet has beneficial cardiovascular effects among individuals with T2DM both through indirect (i.e., the reduction of the body weight and visceral fat in the abdominal area, thus contributing to a reduction in insulin resistance), and direct biological mechanisms (i.e., glucose-lowering effect). Overall, the Med-diet promoted improved glycemic control and exerted preventative effects on the cardiovascular system [30].

Smoking represents one of the most studied high-risk habits and is a well-known risk factor for the occurrence of CVD [31]. In our study, those who smoked were independently associated with a low adherence to the Med-diet. It is possible that this relationship underlies the individual’s overall poor adherence to healthy habits, with potential serious effects on the development and prognosis of CVD. Moreover, the detrimental effects of active smoking and poor dietary habits can be synergistic, and eventually, further accentuated by other lifestyle behaviors. Further knowledge of these complex interplays is pivotal to the implementation of effective strategies for CVD prevention, and therefore warrants further investigation.

Gender, as a determinant of health status, is poorly explored in clinical research [11,32]. The main obstacle in the integration of gender-related factors, in clinical studies, stems from the lack of a standardized and validated measure of gender. The GENESIS-PRAXY study pioneered a methodology for measuring gender using a composite measure of gender in the form of a derived gender score (GPGS) [19]. This gender score was based on a multidimensional evaluation of psycho-socio-cultural factors. Independent from biological sex, the GPGS predicted adverse clinical outcomes among adults with IHD, laying the foundation for the clinical incorporation of a gender-based approach [20,21]. In this light, we hypothesized that adherence to the Med-diet could be influenced by gender-related factors. As expected, in our cohort, we have shown that perceived stress and personality traits have an independent effect on the extent of adherence to the Med-diet. Specifically, male personality traits (including risk-taking, independence, competitive attitude, all items of the male BSRI score), identified that both men and women who are less likely to exhibit low adherence to the Med-diet. These findings provide further enlightenment regarding the influence of personality traits in eating behavior [33]. Some personality traits, evaluated in conjunction with the “big five personality traits”, attributable to male biological sex (i.e., the tendency to take financial or recreational risks and extroversion), have already been associated with a higher consumption of vegetables and fruit, essential items of the Med-diet, in unselected populations [34,35].

The level of perceived stress is another gender-related factor that captures the nuances of gender identity and it was found to be associated with low Med-diet adherence. In the literature, the interaction between stress and dietary patterns has been rarely explored. Prior work has demonstrated that stress mediates the association between loneliness and low Med-diet adherence in a cohort of young adolescents [36]. The intake of food rich in sugar and fats, which is not part of the Med-diet, could serve as an escape and coping strategy to reduce perceived stress [37]. We also observed that adults with IHD and high perceived levels of stress are more likely to have a low adherence to the Med-diet before admission for an ischemic event. This finding supports the detrimental impact of a factor related to gender identity in maintaining a healthy dietary pattern. Therefore, reinforcing the individual ability of coping with stress represents a potential gender-sensitive intervention to target this nontraditional modifiable risk factor and to address adherence to the Med-diet across the lifespan.

The main strengths of the present analysis are the unique granularity of information on the four dimensions of gender in EVA cohort and the real-world data from adults with IHD. We were optimally positioned to test the effect of the individual gender-related factors on participants’ attitudes toward adherence to the Mediterranean healthy dietary pattern.

### Limitations

Some important limitations of the present analysis must be stated. As with any observational cohort study, some confounders could not be assessed and could influence our final multivariate model, as could any missing data points. Furthermore, the sample size was small, and the EVA study was not specifically designed to test the effect of gender-related factors on adherence to the Med-diet. We cannot exclude that, as the sample size increases, some relevant differences could emerge. For example, prior work showed that low socioeconomic status was associated with low Med-diet adherence among a large cohort of Italian healthy subjects [38], while we did not find such association in our cohort of older adults with IHD.

Finally, the results refer to a selected cohort of individuals with high cardiovascular risk, hospitalized for IHD, at a single center in Italy. All these factors contribute to limitations in the generalizability of our results. Nevertheless, our data proposes a unique perspective to a previously poorly investigated issue.

## 5. Conclusions

The present analysis suggests that, in a cohort of adults with IHD, low adherence to the Med-diet implicates various clinical and gender-related determinants. Current smokers and individuals with higher perceived levels of stress are more likely to exhibit a low adherence to the Med-diet, while male personality traits were identified in both men and women with higher a likelihood of being adherent to a healthy dietary pattern, regardless of age and presence of comorbidities.

## Figures and Tables

**Table 1:** Baseline clinical characteristics of (Endocrine Vascular Disease Approach) EVA project participants according to the adherence to the Mediterranean diet.

Variables	Low Adherence (*n* = 81)	Medium-High Adherence (*n* = 285)	*p*-Value
**Age, years (mean ± SD)**	64 ± 13	67 ± 10	0.06
**Women**	31 (38.3)	81 (28.4)	0.09
**BMI, Kg/m^2^ (mean ± SD)**	26.8 ± 4.6	27.2 ± 4.5	0.55
**Previous MI**	22 (27.2)	64 (22.5)	0.38
**Heart failure**	6 (7.4)	37 (13.0)	0.17
**Hypertension**	60 (74.1)	229 (80.4)	0.22
**Dyslipidemia**	35 (43.2)	150 (52.6)	0.13
**Diabetes**	13 (16.0)	77 (27.0)	0.04
**Stroke/TIA**	8 (9.9)	33 (11.6)	0.67
**COPD**	5 (6.2)	29 (10.2)	0.27
**Connective tissue disease**	3 (3.7)	2 (0.7)	0.04
**Active smoking**	30 (37.0)	66 (23.2)	0.01
Physical inactivity*	66 (83.5)	215 (76.0)	0.15
Living with a smoker*	31 (38.3)	83 (29.6)	0.14
**Polypharmacy**	31 (38.3)	124 (43.5)	0.40
**DASI (median (IQR))**	38.2 (24.2–58.2)	36.7 (20.6–50.7)	0.31

Data are presented as number of patients (%), unless otherwise specified. SD, standard deviation; BMI, body mass index; MI, myocardial infarction; TIA, transient ischemic attack; COPD, chronic obstructive pulmonary disease; DASI, Duke Activity Status Index; IQR, interquartile range. *:<10% missing data.

**Table 2:** Angiographic characteristics of EVA participants, according to the adherence to the Mediterranean diet.

Variables	Low Adherence (*n* = 81)	Medium-High Adherence (*n* = 285)	*p*-Value
**Obstructive CAD**	60 (74.1)	202 (70.9)	0.57
**Multivessel CAD**	55 (67.9)	213 (74.7)	0.23
**Presentation as ACS**	46 (56.8)	140 (49.1)	0.22

Data are presented as number of patients (%). CAD, coronary artery disease; ACS, acute coronary syndrome.

**Table 3:** Gender-related factors according to the adherence to the Mediterranean diet.

Variables	Low Adherence (*n* = 81)	Medium-High Adherence (*n* = 285)	*p*-Value
**Married/living with partner**	55 (67.9)	194 (68.1)	0.98
Low social support*	9 (12.0)	21 (7.8)	0.25
**Employment**	36 (44.4)	104 (36.5)	0.19
Household’s primary earner*	35 (45.5)	137 (48.9)	0.86
Low household income*	21 (26.6)	77 (27.5)	0.48
Low education level*	48 (60.0)	156 (54.7)	0.40
**Male BSRI ‡ (median (IQR))**	4.8 (4.1–5.5)	5.1 (4.5–5.6)	0.048
**Female BSRI ‡ (median (IQR))**	6.0 (5.5–6.4)	5.9 (5.4–6.4)	0.66
**Neutral BSRI ‡ (median (IQR))**	5.0 (4.6–5.5)	4.9 (4.3–5.3)	0.21
PSS-10* **(median [IQR])**	19 (11–23)	15 (11–20)	0.07

Data are presented as number of patients (%), unless otherwise specified. BSRI, Bem Sex Role Inventory; PSS-10, Perceived Stress Scale 10 items; IQR, interquartile range. * <10% missing data and ‡ data available for 297 patients.

**Table 4:** Factors associated with low adherence to the Mediterranean diet, evaluated by univariate analysis.

Variables	O.R.	95% CI		*p*-Value
		Lower	Upper	
**Female Sex**	1.56	0.93	2.62	0.091
**Age**	0.98	0.96	1.00	0.032
**Diabetes**	0.52	0.27	0.99	0.046
**Connective tissue disease**	5.44	0.89	33.14	0.066
**Current smoker**	1.95	1.15	3.31	0.013
**Male BSRI**	0.72	0.54	0.97	0.029
**PSS-10**	1.03	1.00	1.07	0.063

OR, odds ratio; CI, confidence interval; BSRI, Bem Sex Role Inventory; PSS-10, Perceived Stress Scale 10 items.

**Table 5:** Factors associated with low adherence to the Mediterranean diet, evaluated by logistic regression analysis, regardless of age, sex, and comorbidities.

Variables	O.R.	95% CI		*p*-Value
		Lower	Upper	
**Current Smoker**	2.10	1.14	3.85	0.017
**Male BSRI**	0.70	0.52	0.95	0.021
**PSS-10**	1.04	1.00	1.08	0.038

OR, odds ratio; CI, confidence interval; BSRI, Bem Sex Role Inventory; PSS-10, Perceived Stress Scale 10 items.

## Appendix A

**Table A1:** Self-administered questionnaire for the assessment of the adherence to the Mediterranean diet (from Martínez-González, M.A.; García-Arellano, A.; Toledo, E.; Salas-Salvadó, J.; Buil-Cosiales, P.; Corella, D.; Covas, M.I.; Schröder, H.; Arós, F.; Gómez-Gracia, E. et al., A 14-item Mediterranean diet assessment tool and obesity indexes among high-risk subjects: The PREDIMED trial. PLoS One 2012, 7).

Questions	Criteria for 1 Point
**1. Do you use olive oil as main culinary fat?**	Yes
**2. How much olive oil do you consume in a given day (including oil used for frying, salads, out-of-house meals, etc.)?**	≥4 tbsp
**3. How many vegetable servings do you consume per day? (1 serving: 200 g [consider side dishes as half a serving])**	≥2 (≥1 portion raw or as a salad)
**4. How many fruit units (including natural fruit juices) do you consume per day?**	≥3
**5. How many servings of red meat, hamburger, or meat products (ham, sausage, etc.) do you consume per day? (1 serving: 100–150 g)**	<1
**6. How many servings of butter, margarine, or cream do you consume per day? (1 serving: 12 g)**	<1
**7. How many sweet or carbonated beverages do you drink per day?**	<1
**8. How much wine do you drink per week?**	≥7 glasses
**9. How many servings of legumes do you consume per week? (1 serving: 150 g)**	≥3
**10. How many servings of fish or shellfish do you consume per week? (1 serving 100–150 g of fish or 4–5 units or 200 g of shellfish)**	≥3
**11. How many times per week do you consume commercial sweets or pastries (not homemade), such as cakes, cookies, biscuits, or custard?**	<3
**12. How many servings of nuts (including peanuts) do you consume per week? (1 serving 30 g)**	≥3
**13. Do you preferentially consume chicken, turkey, or rabbit meat instead of veal, pork, hamburger, or sausage?**	Yes
**14. How many times per week do you consume vegetables, pasta, rice, or other dishes seasoned with sofrito (sauce made with tomato and onion, leek, or garlic and simmered with olive oil)?**	≥2

## Appendix B

List of EVA investigators

**EVA Collaborative Group:** Andrea Lenzi, Claudio Tiberti, Francesca Panimolle, Andrea Isidori, Elisa Giannetta, Mary Anna Venneri, Laura Napoleone, Silvia Quattrino, Simona Ceccarelli, Eleni Anastasiadou, Francesca Megiorni, Cinzia Marchese (Department of Experimental Medicine, Medical Physiopathology, Food Science and Endocrinology Section, Sapienza University of Rome, Rome, Italy); Carlo Gaudio, Enrico Mangieri, Gaetano Tanzilli, Nicola Viceconte, Francesco Barillà, Vincenzo Paravati, Guglielmo Tellan, Evaristo Ettorre, Adriana Servello, Fabio Miraldi, Andrea Moretti, Alessandra Tanzilli, Piergiovanni Mazzonna, Suleyman Al Kindy, Riccardo Iorio, Martina Di Iorio, Gennaro Petriello, Laura Gioffrè, Eleonora Indolfi, Gaetano Pero, Nino Cocco, Loredana Iannetta, Sara Giannuzzi, Emilio Centaro, Sonia Cristina Sergi, Simona Bartimoccia, Antonio Fraioli, Silvia Nocchi, Mario Fontana, Sergio Morelli, Pasquale Pignatelli, Salvatore Minisola, Francesco Violi (Department of Clinical, Internal, Anesthesiological and Cardiovascular Sciences, Sapienza University of Rome, Rome, Italy); Filippo Toriello, Eleonora Ruscio, Tommaso Todisco, Nicolò Sperduti, Giuseppe Santangelo, Giacomo Visioli, Marco Vano, Ludovica Maria Antonini, Silvia Robuffo, Bernadette Corica, Claudia Tucci, Agostino Rossoni, Annarita Vernile, Mariateresa Santoliquido, Verdiana Santori, Giulia Tosti, Fabrizio Recchia, Francesco Morricone, Roberto Scacciavillani, Alice Lipari, Andrea Zito, Floriana Testa, Giulia Ricci, Ilaria Vellucci, Marianna Vincenti, Silvia Pietropaolo, Camilla Scala, Nicolò Rubini, Marta Tomassi, Claudia Ciancarella, Biagio Scotti, Claudio Cantelmi, Floriana Santomenna, Giacomo Costanzo, Gloria Rozzi, Lucas Rumbolà, Salvatore Giarrizzo, Carlotta Sapia, Giuseppina Cusano, Andrea Palladino, Francesca Villani, Antonella Cacciani, Massimo Granata, Lucia Stefanini, Giovanni Talerico, Sebastiano Filetti, Massimo Fiorilli (Department of Translational and Precision Medicine, Sapienza University of Rome, Rome, Italy); Danilo Toni, Anne Falcou (Emergency Department Stroke Unit, Sapienza University of Rome, Rome, Italy); Louise Pilote, Uri Bender (McGill University Health Centre Research Institute, Centre for Outcomes Research and Evaluation, Montreal, QC, Canada); Anna Rita Vestri (Department of Public Health and Infectious Disease, Sapienza University of Rome, Roma, Italy); Patrizia Ferroni (San Raffaele Roma Open University and Inter-institutional Multidisciplinary Biobank, IRCCS San Raffaele Pisana, Rome, Italy); Clara Crescioli, Cristina Antinozzi, Francesca Serena Pignataro (Department of Movement, Human and Health Sciences Section of Health Sciences, Unit of Endocrinology, Università degli Studi di Roma “Foro Italico”, Rome, Italy); Tiziana Bellini, Alessandro Trentini (Department of Biomedical and Specialty Surgical Sciences, University of Ferrara, Ferrara, Italy); Roberto Carnevale (Department of Medico-Surgical Sciences and Biotechnologies, Sapienza University of Rome, Latina, Italy); Cristina Nocella (Department of AngioCardioNeurology, IRCCS NeuroMed, Pozzilli, Italy); Carlo Catalano, Iacopo Carbone, Nicola Galea (Department of Radiological, Oncological and Pathological Sciences, Sapienza University of Rome, Rome, Italy); Giuliano Bertazzoni, Marianna Suppa, Antonello Rosa, Gioacchino Galardo, Maria Alessandroni, Lorena Cipollone (Department of Emergency Medicine, Policlinico Umberto I, Sapienza University of Rome, Rome, Italy); Alessandro Coppola, Mariangela Palladino (Chest Pain Unit, Policlinico Umberto I, Rome, Italy); Giulio Illuminati, Fabrizio Consorti (Department of Surgical Sciences, Sapienza University of Rome, Rome, Italy); Paola Mariani, Fabrizio Neri, Paolo Salis, Antonio Segatori, Laurent Tellini, Gianluca Costabile (Nursing Team Catheterization Lab Policlinico Umberto I, Rome, Italy).
